# The Preventing Australian Football Injuries with Exercise (PAFIX) Study: a group randomised controlled trial

**DOI:** 10.1136/ip.2008.021279

**Published:** 2009-05-27

**Authors:** C Finch, D Lloyd, B Elliott

**Affiliations:** 1 School of Human Movement and Sport Sciences, University of Ballarat, Ballarat, Victoria, Australia; 2 School of Sport Science, Exercise, and Health, University of Western Australia, Perth, Western Australia

## Abstract

**Background:**

Knee injuries are a major injury concern for Australian Football players and participants of many other sports worldwide. There is increasing evidence from laboratory and biomechanically focused studies about the likely benefit of targeted exercise programmes to prevent knee injuries. However, there have been few international studies that have evaluated the effectiveness of such programmes in the real-world context of community sport that have combined epidemiological, behavioural and biomechanical approaches.

**Objective:**

To implement a fully piloted and tested exercise training intervention to reduce the number of football-related knee injuries. In so doing, to evaluate the intervention’s effectiveness in the real-world context of community football and to determine if the underlying neural and biomechanical training adaptations are associated with decreased risk of injury.

**Setting:**

Adult players from community-level Australian Football clubs in two Australian states over the 2007–08 playing seasons.

**Methods:**

A group-clustered randomised controlled trial with teams of players randomly allocated to either a coach-delivered targeted exercise programme or usual behaviour (control). Epidemiological component: field-based injury surveillance and monitoring of training/game exposures. Behavioural component: evaluation of player and coach attitudes, knowledge, behaviours and compliance, both before and after the intervention is implemented. Biomechanical component: biomechanical, game mobility and neuromuscular parameters assessed to determine the fundamental effect of training on these factors and injury risk.

**Outcome measures:**

The rate and severity of injury in the intervention group compared with the control group. Changes, if any, in behavioural components. Process evaluation: coach delivery factors and likely sustainability.

In 1994, the Commonwealth Government of Australia released its health strategy policy and national health goals and targets.^1^ The injury prevention/control strategy recognised sports injuries as a significant public health problem and a major barrier towards participation in sport/physical activity. Sports injury prevention has also been addressed in other Australian national public health frameworks for both physical activity promotion and sports safety.^2^ ^3^ For example, the 1997 *SportSafe Australia* framework^3^ recognised sports injuries as a potential adverse outcome of increased physical activity participation and emphasised their public health burden for both individuals and society with respect to the duration/nature of treatment, the amount of sport/working time lost, permanent damage/disability, reduced quality of life and other monetary costs.^3^ The most recent estimate of the annual cost of sports injuries to Australians is AUS$1.83 billion.^4^

Importantly, sports injuries are not an inevitable part of participation,^3^ and much can be done to prevent them and reduce their burden on Australian society.^2^ ^3^ ^5^ Despite this, a recent overview of the status of sports safety action in Australia concluded that the evidence base for the effectiveness of a full range of prevention measures is largely non-existent.^2^

## FOOTBALL INJURIES AS A PRIORITY PUBLIC HEALTH ISSUE

This project focuses on preventing knee injuries in Australian Football because it is one of the most popular team sports in Australia (with large numbers of both formal and informal community participants, including females). The Australian Bureau of Statistics has estimated that ∼2% of the general population and 3% of the male population participate in Australian Football.^6^ These figures rank Australian Football as the 13th most popular physical activity with respect to participation rates.

Unfortunately, Australian Football also has a high excess risk of injury. Epidemiological studies of medically treated sports injuries in Australia have consistently shown that Australian Football ranks as the sport associated with the highest number of presentations for injury treatment, accounting for more than 23% of all cases presenting to sports medicine clinics, emergency departments and general practice clinics.^7^^–^11 Moreover, Australian Football is ranked in the top 10 sports activities leading to injuries that are so severe they require admission to hospital (after an emergency department visit) in Australia.^8^ The prominence of Australian Football in the injury statistics remains even after participation rates are taken into account.^7^ ^11^

Australian Football is a contact sport and running game that involves periods of continuous physical activity, interspersed with periods of high-intensity activity, including unexpected, explosive and agile movements and heavy physical contact. These game features contribute to the high risk of injury. Although preventive measures have been adopted to varying degrees, the current lack of an evidence base limits the safety advice that can be given to players, their parents, coaches and sports administrators about the best injury prevention measures to adopt, while playing Australian Football.^2^

## PREVENTION OF KNEE INJURIES

From a public health point of view, Australian Football-related knee injuries are of particular concern because of their high frequency, associated treatment/rehabilitation costs, absence from participation and other long-term sequelae. Knee injuries account for >30% of all medically treated Australian Football injuries^9^ ^11^ ^12^ and are the most expensive to treat and rehabilitate, especially if they require reconstructive surgery. On the basis of the best available data, we estimate that, if 50% of Australian Football-related knee injuries could be prevented, the annual savings to the Australian community would be of the order of $60 million.

Knee injuries are defined as those to the ligaments surrounding the knee joint. This excludes injuries to the patella and surrounding muscles, eg, the hamstring, but does include tears or partial/full ruptures of the anterior cruciate ligament (ACL) and medial cruciate ligament, meniscal tears and other ligament strains. Ligament injuries are essentially a neuromuscular and biomechanics problem, in that ligaments fail when applied physical loads exceed tissue strength. The critical factor in lowering the incidence of knee joint ligament injury is thus reduction in tissue loading.^13^

Previous research has shown that manoeuvres common to Australian Football, such as running and side stepping, can place large external loads on the knee joint^14^ ^15^ and have the potential to cause injury.^16^ ^17^ Ligament loading has been found to depend on: (*a*) the external loads applied to the joint; (*b*) the joint posture when these loads are applied; and (*c*) the ability of muscles surrounding the joint to reduce ligament loads.^13^^–^18 Alterations in neuromuscular and biomechanical factors can reduce ligament loading and reduce the potential for non-contact ligament injury by: (*a*) reducing the external loads applied to the joint; (*b*) changing knee joint posture and motion to reduce load; (*c*) improving the muscles’ abilities to counter large loads; and/or (*d*) improving the ligaments’ ability to withstand large loads.

It has been proposed that the most appropriate intervention strategy to achieve these neuromuscular and biomechanical changes is a well-designed exercise programme.^19^^–^21 Prior research provides the rationale for training programmes (including skill-based, balance and perturbation) to reduce ACL loading and therefore risk of injury in Australian Football.^19^ These intervention strategies include plyometric, perturbation and skill training as well as traditional proprioceptive training. Specific technique factors involved in performing sporting actions to decrease ACL loading and risk of injury have been identified.^14^ ^15^ ^22^ These theories have been confirmed in a randomised controlled trial (RCT) of 50 elite footballers: an aggressive balance-training programme was shown to (*a*) increase the level of hamstrings and quadriceps co-contraction and (*b*) reduce frontal plane knee loading by 35%. Both of these adaptations would reduce knee ligament loading and theoretically lower injury risk.

Although preventing sports injuries has been defined by the Australian National Health and Medical Research Council (NHMRC) and others as a priority research area, the effectiveness of exercise training programme modalities to prevent knee injuries in Australian Football, and indeed in most other sports, have not previously been undertaken.^2^ ^5^ Although it is common for specific exercise programmes to be undertaken by high-level/elite Australian Football players, these are generally promoted as a means of performance enhancement and have not been widely adopted at the broad community level of play. Furthermore, some of the more traditional “off-field” training regimens for community sport may actually increase knee loading in Australian Football game movements.^22^ ^23^ There is a particular need for the development of exercise training programmes for community-level players, as they have a high risk of knee injury and the focus of their involvement is continued safe and healthy participation and not performance.^2^

## RATIONALE FOR COMPONENTS OF AN EFFECTIVE EXERCISE TRAINING PROGRAMME

As jumping and landing manoeuvres have been implicated in causing ACL injuries, plyometric training (which involves jumping, bounding and landing to improve performance in explosive manoeuvres) has the potential to lower the risk of injury. Indeed, both this training and increasing jumping and bounding performance have been used to reduce the incidence of knee injuries in young female athletes.^24^ There is also some evidence that traditional proprioceptive training can reduce the incidence of ACL injuries.^20^ This training places demands on both whole-body balance and joint stability and can alter the voluntary and reflexive muscle activation patterns that may be in effect during sporting actions and potentially reduce the loads placed on the ligaments of the knee.^18^ ^22^ ^25^ ^26^ Recent research has elucidated the neuromuscular adaptations that stem from proprioceptive training^18^ ^25^ ^27^ and how these changes decrease the risk of ACL injury through reducing the loading of knee ligaments.^14^

Another exercise modality, whole-body perturbation training, is beginning to receive attention for its effect on neuromuscular mechanisms that enhance joint stability and decrease ligament loading.^19^ ^21^ ^28^ For example, whole-body perturbations applied to ACL-deficient subjects have been shown to improve dynamic knee joint stability and function and to reduce clinical symptoms.^21^ Knee-specific perturbation training which stimulates ligament mechanoreceptors in the knee could enhance voluntary movement control to improve joint stability and reduce ligament loads and injury risk.^19^ ^21^ ^28^

Skill training involves providing players with a qualitative description of techniques that will minimise their risk of injury while performing specific sporting movements (eg, cutting and landing). Changing torso movement relative to foot placement, lowering body centre of mass, increasing knee flexion during ground contact and increasing hamstring/quadriceps co-contraction during pre-foot contact and early and mid stance are technique modifications that have been shown to reduce frontal plane knee loading^14^ ^15^ ^22^ and loading on the ACL.^17^ ^19^ ^29^ Cowling *et al*^30^ have shown that it is possible to learn to use increased knee flexion angle when landing using verbal feedback. Other work has also shown that performing sporting tasks in an unanticipated manner increases external knee loads and compromises activation patterns that stabilise the knee and reduce ligament loading.^18^ Skill training should therefore include drills that ensure players are familiar with making “unanticipated” or “spur of moment” side steps.

This paper describes the aims, study design and methodology of the Preventing Australian Football Injuries with eXercise (PAFIX) Study. This study was designed according to the CONSORT statement.^31^ It is being conducted over the 4-year period of 2006–2009. The study is funded by a nationally competitive research project grant from the (Australian) National Health and Medical Research Council.

## RESEARCH AIMS AND HYPOTHESES

The PAFIX project aims to:

1. Reduce the number of Australian Football-related knee injuries using appropriate intervention strategies
2. Evaluate the effectiveness of exercise intervention strategies targeting neuromuscular and biomechanical variables for reducing injury rates

In so doing, it proposes to formally evaluate the effectiveness of exercise training programmes (a widely promoted injury prevention measure) to prevent knee injuries in community-level footballers.

Specific hypotheses are:

1. The rate of knee ligament injuries will be reduced after a targeted exercise training intervention
2. Neuromuscular and biomechanical variables measured preseason will be related to injury risk
3. The neural and biomechanical adaptations resulting from the exercise training intervention will be associated with decreased risk of injury

On the basis of previous laboratory studies undertaken by us and referenced elsewhere in this paper, it is further hypothesised that these injury reductions will be related to the following changes after the specific training programmes:

1. The kinematics during cutting and landing tasks will be different pre to post training in the intervention group, compared with controls
2. Knee joint loading will be reduced pre to post training in the intervention group, compared with controls
3. Mobility in the intervention group will be improved after training, and compared with controls
4. The co-contraction of knee muscles during cutting and landing actions will be increased pre to post training in the intervention group, compared with controls
5. Neuromuscular measures will improve in the intervention training group pre to post training, and compared with controls
6. These neuromuscular and biomechanical changes are related to a reduced risk of knee injury

## THE INTERVENTION BEING TRIALLED

As mentioned above, the intervention being trialled has previously undergone extensive laboratory and other pilot testing. Teams of Australian Football players are randomly assigned to either a control or intervention arm. The training intervention is a combination of skill-based training and perturbation training.^27^ The interventions are based on prior work which has elaborated and reviewed the essential features of, and rationale for, training programmes.^19^ ^27^ For example, some of the intervention components were trialled by us in a small-scale RCT with sub-elite Australian Football players: machine weights, free weights, aggressive balance training and combined machine weights and balance training. Eighty male players were randomly assigned to each of the four training and single control (no training) groups. The neuromuscular biomechanical tests applied before and after training were those previously published^14^ ^15^ ^18^ ^32^ ^33^ and incorporated side-stepping tasks that cause ligament injury in sport. Only the aggressive balance training, a major component of the intervention being trailed in this study, produced changes to reduce ACL loading in side-stepping tasks. These included an 18% increase in knee range of motion in the weight-acceptance phase of stance, a 35% reduction in the varus–valgus loading on the knee, and a 15% increase in the level of co-contraction of the hamstrings and quadriceps muscles.^23^ ^34^ Theoretically, these changes could reduce load on the ACL, thereby translating to a reduction in ACL injury risk. However, the only way to test this hypothesis is to examine the intervention in a larger combined epidemiological and biomechanical study, as is being undertaken here.

### Trial study arms

#### Control arm: control group (normal training programme)

At the community-level of play, most footballers undertake regular training sessions to prepare for the weekend games (matches). These sessions focus on game technique for performance purposes and to enhance the level of game skill, fitness, strength and conditioning. Typically these training programmes do not focus on specific exercises to reduce the risk of injury. Such training programmes are being used by the teams assigned to the control group. Control teams are not provided with any information about how to enhance these training programmes for injury prevention gains. A “sham” intervention has been given to the control teams in arm 1, and this is derived from what has been common practice for the teams plus a few extra exercises that are biomechanically distinct from those included in the intervention arm.

#### Intervention arm: normal training programme + intervention training programme

In addition to their usual training regimens, intervention teams are receiving a combination of skill and perturbation-type exercise training. This programme incorporates two general training modalities: (*a*) perturbation balance training and (*b*) training of technique to perform side-stepping, crossover and landing actions.^15^ ^25^ ^27^ In balance and perturbation training, the task difficulty progresses from standard proprioceptive training to performing sporting actions (eg, kicking, hand-balling) while being perturbed by the trainer. The purpose of perturbing a player is to stimulate the knee ligament mechanoreceptors, enhance voluntary movement control leading to improved joint stability, reduce ligament loads and reduce the risk of injury.^15^ ^19^ ^28^ The perturbation-training programme involves a circuit arrangement to minimise the amount of equipment required. Each circuit station includes a different item of equipment such as a mini-trampoline, aerobic steps, tilt boards, round and square wobble boards, roller boards, Dura discs and Swiss balls. There are different exercises to perform at each station, with the exercises becoming progressively harder as the player becomes more competent. Typical progressions at each station include: performing the exercises with eyes open to eyes shut; doing exercises with no external force being applied; catching and passing a ball; performing exercises while the board or person is being pushed. Most exercises progress from balancing on two feet, to balancing on one foot, to retaining balance while performing movements such as squats. All items of equipment used in the intervention training programmes are currently available from sports equipment suppliers and are relatively inexpensive (eg, wobble boards cost about AUS$40.00) and so are not prohibitive for uptake by community-based sports teams.

It is known that side-stepping techniques lower knee ligament loading and therefore the risk of injury.^14^ ^15^ ^22^ Individual player side-stepping techniques are videotaped and analysed using SiliconCoach software. This software has graphic tools that enable correct and incorrect techniques to be emphasised. Using processed video data, players are taken slowly through each action, identifying and practising the important factors of the technique that must be implemented to make the action safer. Techniques are first practised with the player knowing which actions are to be carried out to achieve the correct technique. Then actions are performed in situations where players do not know which direction or when the action is to be performed until just before execution when instructed by a trainer. As the player becomes more proficient, they progress from performing actions in isolation to performing actions against other players in a simulated match environment. All exercises and their progressions concentrate on working the muscles surrounding the knee joint, improving these muscle activation patterns to increase stability of the knee, and increasing knee flexion angle during task performance. A common method used in knee rehabilitation is self-palpation of the muscles, so that players are familiar with activating specific muscle and therefore learn how co-contraction of muscles “feels”, so that they can practise these actions in “real-life” sporting tasks just before heel strike. Obtaining increases in knee flexion angles during all tasks is facilitated by simple verbal instructions.^30^

## THE RCT METHODOLOGY

The study is a cluster-randomised RCT evaluation of the exercise training programmes in the field over two playing seasons. The cluster randomisation design is the optimal one for implementation in the field. Australian Football is the sport of choice because of its high public health priority ranking for sports injury prevention action and the fact that all of the proposed epidemiological, fieldwork and laboratory-based methodologies have all been fully trialled in this sport. Teams of Australian Football players from community-level Australian Football competitions will be invited to participate in the trial because they provide a natural grouping of players. Furthermore, injury interventions that are adopted into Australian Football practice will need to be implemented at the team level.

The field-based epidemiological methodology has already been pilot tested, adopted in other sports injury prevention RCTs and prospective cohort studies, and published in the international peer-review literature.^11^ ^12^ ^35^^–^43 Injury surveillance and detailed monitoring of exposure (ie, Australian Football participation) and compliance (with each intervention) are undertaken using the previously reported epidemiological methods.^11^ ^12^ ^35^ ^38^ ^41^^–^43 The study also includes a detailed evaluation of player attitudes, knowledge, behaviours and compliance, both before and after the intervention is implemented on the basis of similar studies.^44^^–^50

The proposed RCT is a multi-site study, being conducted in both Ballarat (Victoria, Australia) and Perth (Western Australia). It has purposely been designed as a multi-state trial because there will be fewer control and training teams in each state, leading to a reduced risk of contamination across study arms, ie, controls finding out about the training programmes for the training intervention groups. Although ground conditions appear to be related to injury risk in elite players, no study has yet explored this for community-level players. A further advantage of our two-state study design is that the different ground conditions in the states will enable an assessment of the possible crossover effect of training and ground conditions.

### Recruitment

With the assistance of the parent Australian Football bodies, we identified local Australian Football leagues and their contact details of all suitable clubs/teams. The teams were randomly ordered and then invited to participate in the study until the quota was reached, with the restriction that, overall, 50% of the teams were from Ballarat and 50% from Perth. In agreeing to participate in the study at this stage, no team was informed to which arm of the RCT they would be assigned. Once teams had expressed an interest in participating in the trial, an attempt was made to recruit all players from these teams; informed consent was obtained from them. All players are aged ⩾16 years. When team recruitment was complete, whole teams were randomised to the two study arms in each state. Study teams were recruited into the larger study, even if some players did not give their informed consent for us to monitor them. This recruitment methodology has previously been used in another sports injury prevention RCT and shown to lead to unbiased recruitment of teams and players.^37^ ^41^ ^51^

### Sample size

The intervention is being implemented during training sessions and its effects measured during games. A team consists of 18 members and a game lasts ∼100 min (four quarters of ∼20 min plus ∼5 min time added on). Over a 6-month playing season, each team has ∼20 games and so each team will contribute ∼600 game-hours of exposure to knee injury risk. We used a standard cluster randomised trial sample size calculation for comparing rates between a control arm and an intervention arm.^52^ The knee injury rate in the control arm was estimated as 20 per 1000 game-hours. This figure is based on the observation of 60.2 injuries (of all kinds) per 1000 game-hours (from data from Finch *et al*^11^) and an assumption that approximately one-third of Australian Football injuries involve the knee. To have 80% power to detect a reduction in the injury rate of 35% (ie a rate ratio of 0.65) in the intervention arm compared with controls, we would require 8.8 teams in each arm. This calculation does not take account of any clustering effects of injuries by team. From the same data,^11^ we estimate that the coefficient of variation of team injury rates will be 0.35 (ie, standard deviation of team injury rates divided by mean of team rates). Assuming that the clustering effect will be identical in the intervention arm, the number of teams required must be multiplied by 2.27, ie, 8.8×2.27 = 20 teams per study arm over the 2 years. Thus 20 teams (10 in Ballarat, 10 in Perth) will be randomly allocated to each arm of the study across the two playing seasons.

For continuous biomechanical outcome data measurements, we plan to sub-sample six players per team. Given 20 teams per study arm, this will give us 80% power to detect effects of size (*a*) 0.44 or (*b*) 0.63 standard deviations (SDs) between study arms assuming a clustering effect described by an intracluster correlation coefficient of (*a*) 0.1 or (*b*) 0.4. The SDs refer to total player-to-player variability in the biomechanical outcome, and the intracluster correlation coefficient is the ratio of between-team variability to total variability. To achieve six players with complete biomechanics data, we will enlist eight, expecting ∼20% dropout based on our previous training study. These sample size calculations also provide a conservative lower bound for power for the analysis of attitude scores, as they were based on six players per team, and the change in attitude scores will be available for closer to 18 players. The study will therefore have >80% power for detecting changes in neuromuscular, biomechanical and attitude parameters as described by an effect size of 0.44 SDs.

### Primary data collectors

All field work procedures are rigidly standardised and based on methods previously developed, piloted and used in similar Australian football studies.^11^ ^35^ ^36^ ^38^ ^41^ The good to high reliability and validity of these data collection methods has already been demonstrated.^36^ ^38^ The day-to-day conduct of the RCT is being coordinated by a project manager in each state.

The appointed project managers train and coordinate the activities of a nominated person within each team (ie, the primary data collector or PDC), who is responsible for data collection on site. The PDC is also the team’s exercise trainer for training sessions and game, which has been a major incentive to attract teams into the study and ensure compliance. It is their role to ensure that the RCT training intervention is run in cooperation with the team coaches, and to ensure that players are compliant and progress to more advanced training tasks as they become more proficient.

It was compulsory for all PDCs to attend a separate formal training session for the intervention arm that covers all study procedures. A detailed procedures manual was disseminated to all PDCs and the clubs they are associated with; these contain the standardised data collection forms for recording exposure, compliance and injuries as well as full details of the coding and classification schemes. During the training sessions, practice exercises on specific examples were undertaken to address any discrepancies before the data collection period starts.

Previous research has demonstrated the use of PDCs in this context as a valid and reliable means of exposure, compliance and injury data collection.^36^ ^38^ ^41^ Club-based PDCs have the particular advantage of knowing all members of the team well and being able to determine very quickly and accurately if a player is missing from a game or training session and determining the reasons why (eg, related to injury?). The accuracy of the PDC activities is being assessed through double counting of exposure and injury data at randomly selected games.^38^ The work being undertaken by the PDCs is not very different from standard practice in Australian Football clubs, which already typically involves the recording of the team members present and any injuries that occur.

### Injury and exposure details

The PDCs record the occurrence of all injuries and their circumstances, and provide injured player details for each game and training session using previously used standardised methods.^12^ ^35^ ^41^ A standardised form is used to provide a simple description of injury and notification that the player was seen by a doctor or sent to hospital. This information is obtained from the PDCs each week.

A self-report injury diagnosis will be obtained from the player after they have sought medical or other treatment for that injury. The injured player will also be asked to provide the details of the person who provided their treatment, so that their self-report information can be validated and full details of the diagnosis obtained from their medical practitioner. Medical records pertaining to injuries sustained during the games will also be obtained, and the injury descriptions reviewed by an associate investigator with medical qualifications. A medical diagnosis will be obtained and coded for each case, and this will be checked with the original notification of injury.

For each player, the amount of time spent playing Australian Football (both competition and training) over each week of the trial is recorded by the PDC. This is done in accordance with the methods previously developed for other community-based Australian Football injury studies.^12^ ^35^ ^41^ ^43^ Exposure is recorded during each training session and game.

### Biomechanical, neuromuscular and mobility outcome measures

The neuromuscular and biomechanical changes induced by the various aspects of the training programmes have already been demonstrated in elite footballers. It is, however, important to assess the effectiveness of the training programme on adaptations and injury rates in community-based footballers. Theoretically, these changes should reduce ligament loading and the risk of injury. However, before they can be readily implemented, it is essential to see if these changes are indeed associated with reduced injury rates. This combination of results will provide confidence in the theoretical basis for the laboratory-based studies used to investigate injury mechanisms and reduction.

To this end, biomechanical measures related to reduction in knee ligament loading will be collected from a subset of players as mentioned above. These measures will be taken while the players perform cutting and landing tasks and entail: (*a*) knee flexion angle at heel strike; (*b*) frontal plane kinematics (position and movement) trunk and pelvis position relative to foot; and (*c*) hamstrings and quadriceps muscle activation patterns. Neuromuscular measures to be taken are: (*a*) knee movement sense and position sense; (*b*) time to generate maximum knee extension and flexion torque in response to a light stimulus; (*c*) isometric knee extension and flexion strength; and (*d*) standing balance. Finally, mobility in the game situation will be assessed by (*a*) agility cone test and (*b*) reaction time to perform the cutting manoeuvres in response to a light stimulus. This battery of tests will be performed on eight players randomly selected from each team in the RCT arms in Perth only and will be taken before training starts and half way into the season. All tests will be performed in the Sports Motion Analysis Laboratory in the School of Sport Science, Exercise and Health at UWA. The cutting and landing tasks will be collected using a 12-camera Vicon 612 system with two force plates to measure kinetic data and a 16-channel telemetry electromyographic system to measure muscle activation patterns. Each participant will perform a series of trials for each task as per the procedures already developed.^14^ ^15^ ^18^ ^19^ Mathematical optimisation algorithms with a cluster marker set have already been developed to improve the repeatability of biomechanical data,^14^ ^15^ ^53^ in some cases by as much as much as 100%.^53^ The neuromuscular parameters assessed in this study will be acquired and processed using established procedures^14^ ^18^ ^32^ ^33^ that are currently used in this laboratory.

In Ballarat, we intend to use some of the neuromuscular and mobility tests selected from the battery of tests performed in Perth. These selected tests will be those that can easily be performed in the field situation. These will be: (*a*) knee movement sense and position sense; (*b*) isometric knee extension and flexion strength; (*c*) standing balance; (*d*) agility cone test. These will also be performed on eight randomly chosen people from each team and taken before training starts and about half way into the season.

### Blinding of interventions

To the extent possible, the interventions have been blinded. All teams/players were told that they are involved in an assessment of the value of their training programme. No team was told to which arm of the trial they had actually been allocated nor were they specifically told that the outcome of interest is knee injury prevention. All players are required to adopt their allocated exercise training programme throughout the playing season.

As the study is being conducted in two states, the study teams are being drawn from a large base and therefore we do not expect contamination of the study arms. The club-based PDCs are unaware of the study design as a two-arm RCT and thus operate independently believing that the study is investigating the relationship between their training programme and injury profile. Thus, the monitoring of injuries and player exposure is independent of, or blinded to, the other interventions.

### Adjustment for, and strategies to overcome, potential confounding

A possible confounder of the relationship between the training programmes and injury outcomes are the ground conditions, particularly as the RCT is to be conducted in two different states. Research on injury in elite Australian Football players has identified ground conditions as an important factor,^54^ but this has not been explored in community-level players. Using previously published methods, a ground conditions report is obtained for all matches. This includes assessment of (*a*) soil and grass type (obtained from the groundsperson and classified as mainly couch grass, mainly rye, etc and natural sand base, sandy loam, etc) and (*b*) rainfall reading on the day of the game (obtained from a nearby weather station). If the Australian Football grounds are close to where rainfall is recorded by the Bureau of Meteorology, then it is easy to collect daily rainfall data for up to a year before.

Information on additional exercise undertaken by players is collected directly from players at both preseason and end-of-season surveys. This includes information on the type, frequency and intensity of such exercise and will be adjusted for in the analysis. Any additional exposure is expected to be evenly distributed across the two arms; therefore there is very little risk of non-differential misclassification of exposure.

### Behaviour, attitudes and knowledge

In addition to the RCT, the attitudes, knowledge and behaviours of the players with regard to the interventions are being determined both before and after the interventions are implemented. This has been undertaken through surveys of all players involved in the RCT both preseason and postseason using a standard questionnaire, based on those developed previously^39^ ^44^ ^45^ ^47^ ^49^ and including measures of risk, playing and injury history and basic sociodemographic profile. The surveys assess, in particular, whether players in different study arms hold different risk perceptions and modify their behaviours accordingly, and whether these change over the seasons.

### Compliance

This study has been optimally designed to maximise compliance in a community sport setting. However, this aspect is being monitored vigilantly throughout the study and any contamination in the control arm will be adjusted for in the final analysis.

The coach of an Australian Football team, in conjunction with the referee, has jurisdiction over the behaviour of his/her team’s members and plays a very important role in this by ensuring that players comply with the interventions. This is standard Australian Football practice and will be used to the projects’ full advantage.

The PDCs record whether or not players are compliant during each trial week on a data record sheet. These data will be combined over all exposure episodes, and an index of compliance (such as percentage of exposure episodes that were compliant) will be computed for each player. Experience from previous work^37^ ^51^ ^55^ has shown that coaches do support injury prevention research and are particularly supportive of interventions such as those proposed here, which could also enhance their team’s performance.

The project is being conducted at the upper levels of community Australian Football—that is, division 1 and A grade amateur players. As players and coaches from these levels are more motivated to play well, and therefore more likely to be motivated to undertake training programmes, that could help this.

### Analysis

At the start of the RCT, retrospective injury data are obtained to determine equivalence in injury risk at baseline. Injury, exposure and compliance data are being collected during each month of the Australian Football season, and the outcome measures of interest are the total numbers of injuries, total exposure and overall compliance rates over the entire study period. All data will be pre-coded and double-entered into a PC database.

All analyses will be undertaken with the Stata analysis package and performed on an intention-to-treat basis with the control arm (arm 1) as the reference group. The analysis of biomechanical outcomes will take clustering of individuals within teams into account through the use of generalised estimating equations. Poisson or negative binomial regression, as appropriate, will be used to analyse injury rates with estimation of a scale parameter allowing for cluster effects.

Behavioural, attitudinal and knowledge variables will be assessed by a number of different items on a questionnaire. Responses will be scored, and the scores from different items combined to give an overall attitude score on a quasi-continuous scale. Scores will be compared preseason to postseason, and changes compared across study arms. The attitudes and behaviours of players, particularly attitudes before and after, will be compared by categorical data analysis again using generalised estimating equations to allow for cluster effects. Information about injury outcomes (ie, time away from sport and amount of treatment received) will be collected from all injured players so that the cost per injury can be calculated.

### Project time frame

The project is being conducted over the 4-year period of 2006–2009 (including two playing seasons) as shown below. Given the timing of the Australian Football season, and the need to produce the training instruction kits, data collection forms, and to set-up the biomechanical testing procedures, the trial phase itself did not start until year 2.

- Jan–Mar 2006: Staff advertising and appointment
- Mar–Dec 2006: Preparation of data collection forms/setting-up of biomechanical field testing procedures/production of training instruction kits/pilot testing of attitudinal survey procedures
- Jan–Mar 2007 (and Jan–Mar 2008): Recruitment of teams and players/randomisation/training of PDCs
- Mar–Apr 2007 (and Mar–Apr 2008): Attitudinal surveys
- Mar–Aug 2007 (and Mar–Aug 2008): Implementation of interventions/injury surveillance/exposure monitoring/compliance checks/biomechanical studies
- Sep–Dec 2007 (and Sep–Dec 2008): Data entry/database management/preliminary data analysis
- 2009: Data analysis and publication writing

## OUTCOMES AND SIGNIFICANCE

Sports injury prevention efforts to date in Australia have been severely hampered by the lack of evidence supporting currently advocated countermeasures. This concern has been recognised by the NHMRC. It is now time to implement and formally evaluate the effectiveness of sports injury countermeasures in the context of broad community level participation in sport, in contrast with previous research focused on professional sport, and deliver the preventive measures to the community. Focusing attention on demonstrating the effectiveness of Australian Football injury interventions in the field will establish Australian leadership in sports safety research and assist the delivery of safe sport in Australia. Sports participants will benefit through safer sporting environments that ensure that maximum participation and its associated health benefits can be achieved over a full lifespan.

In order to progress sports safety efforts in this country, it is important to demonstrate that the implementation of any sports safety strategy, such as exercise training regimens, achieves its desired objectives (ie, increased adoption rates and reduced injury rates). In addition, any unexpected adverse effects of safety measures, eg, any changes in participation rates and player attitudes and/or behaviours need to be monitored. To date, there have been very few internationally published formal evaluations of any sports injury prevention measure such as training programmes. From a public health perspective, it is necessary to act at the broad community level of play because of the large numbers of participants at these levels. National, state and local sports bodies can only begin to develop and implement “best practice” injury prevention when they can identify injury priorities and effective strategies for their prevention, based on a firm evidence base. Rigorous research and evaluation of effective countermeasures is therefore required to progress sports safety in Australia. A major strength of this project is the application of fully piloted basic laboratory work to the real-world setting to address a problem of public health significance through the uniting of epidemiological and biomechanical approaches.

Most knee injuries (over 60%) in community-level Australian Football are not associated with body contact. Our interventions have the potential to reduce the incidence of both contact and non-contact injuries, because they will improve the knee’s resistance to forces placed on the lower limb joints during such contact. All injuries, irrespective of their body site or cause, will be recorded in players from each RCT arm so that this can be ascertained.

For logistical reasons, this study is being conducted on Australian Football players only. However, it has significant potential to inform injury prevention efforts in other sports with a significant risk of knee injury (eg, women’s netball). It will do this in four ways.

1. Few large-scale RCTs of knee injury prevention exercise programmes in any sport have been previously reported in the international literature; this project will therefore inform the design and conduct of future studies.
2. It will provide valuable guidance on how to work with sporting bodies, coaches and players to maximise the uptake of injury interventions.
3. The training programme is a general one which could, should it prove effective, be immediately applied to other sports.
4. It will provide further confidence in linking the results of laboratory-based biomechanics injury research to large-scale community-based RCTs, thus supporting this research model to produce solutions to injury problems.
